# QSAR Study on a Series of Protein Tyrosine Phosphatase 1B Inhibitors

**Published:** 2008-12

**Authors:** Rajendra S. Mehta, Hetal R. Prajapati, Dinesh V. Thakkar, Pathik S. Brahmkshatriya

**Affiliations:** 1 *Department of Pharmaceutical Chemistry, A R College of Pharmacy and G H Patel Institute of Pharmacy, Mota Bazar, Vallabh VidyaNagar, Dist. Anand, Gujarat, India;*; 2 *Department of Pharmaceutical Chemistry, L M College of Pharmacy, Navrangpura, Ahmedabad, Gujarat, India*

**Keywords:** diabetes, PTP1B, QSAR

## Abstract

As a therapeutic target, protein tyrosine phosphatase 1B (PTP1B) has received considerable attention for the treatment of diabetes mellitus. A QSAR study using substituted monocyclic and polycyclic thiophene derivatives, recently reported as potent PTP1B inhibitors, was carried out. More than 60 physicochemical descriptors were calculated which underwent rational selection before their use in derivation of QSAR models. Statistically significant equations were generated using multiple linear regression analysis. External validation of the derived models with test set compounds proved good predictability of the models. Interpretation of the results revealed lipophilicity as a key regulatory feature which affects PTP1B inhibition along with several electronic and steric parameters. The study provides an important platform upon which novel rationally designed molecules can be synthesized with cautious optimism.

## INTRODUCTION

Type-2 diabetes mellitus is the only non infectious disease recognized as epidemic by WHO because of its worldwide diffusion especially in western lifestyle countries (1). It is characterized by chronic elevated blood glucose levels. The increased incidence of type-2 diabetes mellitus and obesity in the population has fueled an intense search for new therapeutic treatment options (2). Resistance to the hormone insulin in the muscle, liver and in central nervous system (3, 4) is characteristic of both type-2 diabetes (1) and obesity (5). Drugs that can ameliorate this resistance should be effective in treating this disease. Protein tyrosine phosphatase (PTPase) catalyzes the removal of phosphate group from phosphotyrosil residues in many proteins.

PTP1B is the first purified PTP (6) and has been demonstrated to dephosphorylate the insulin receptor and thereby attenuate the tyrosine kinase activity, thus acting as negative regulator of insulin signaling (7, 8). A recent study using PTP1B knockout mice showed increased insulin receptor phosphorylation and enhanced sensitivity to insulin in skeletal muscle and liver. In addition, PTP1B knock out mice have remarkably low adiposity and are protected from diet induced obesity (9, 10). Most importantly these mice appeared to be normal and healthy, which indicates that specific inhibitors would be free of side effects and have selective therapeutic efficacy. Thus, PTP1B has become an attractive therapeutic target for the treatment of type-2 diabetes and obesity. A number of research groups have developed small molecules targeting this enzyme (11-16).

Quantitative structure activity relationship (QSAR) plays an important role in lead structure optimization. QSAR methods attempt to capture the relationship between structural features of molecules and their biological activities. Among the several PTP1B inhibitors described in the literature, mono, bi and tricyclic thiophene derivatives present interesting small molecule targets for drug design due to their synthetic accessibility and high potency (17-19). In the present study, a data set of 33 compounds, considered to be PTP1B inhibitors, was used to develop 2D QSAR models to help optimizing the lead from the derived information.

## MATERIALS AND METHODS

### Data set

All calculations were carried out on a windows based PC workstation, using the software package Chemoffice. A series of 33 compounds reported by Wan Zhao-Kui *et al* (17-19) were used for the present QSAR study. The chemical structures and biological properties for the complete set of compounds are listed in Table 1-4. The data set was divided into a set of 27 training compounds (compound 1-27) and 7 test compounds (compound 28-33).

**Table 1:** Structure and biological activity of compounds 1-11 
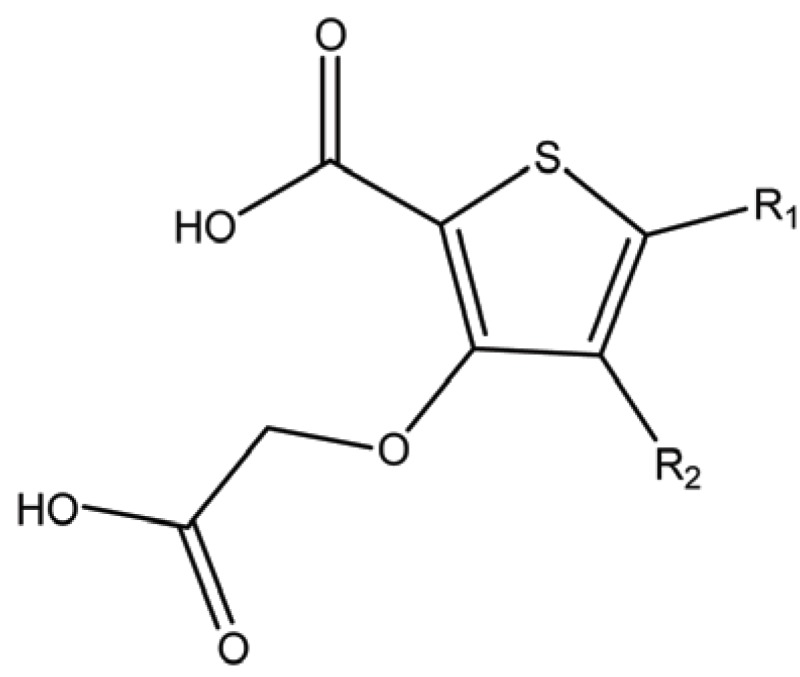

Compd. No.	R_1_	R_2_	Ki (μM)

**1**	Br	Br	18
**2**	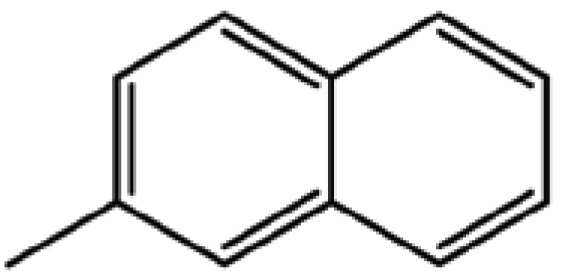	Br	3.3
**3**	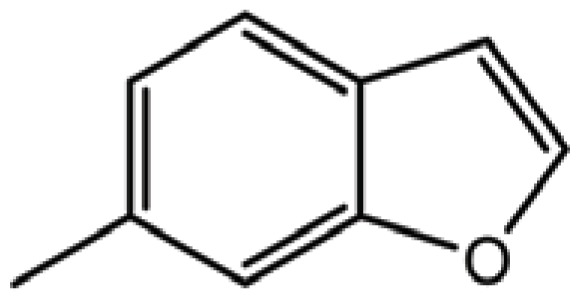	Br	5
**4**	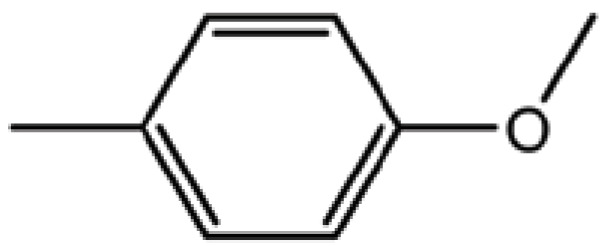	Br	3
**5**	H	Br	160
**6**	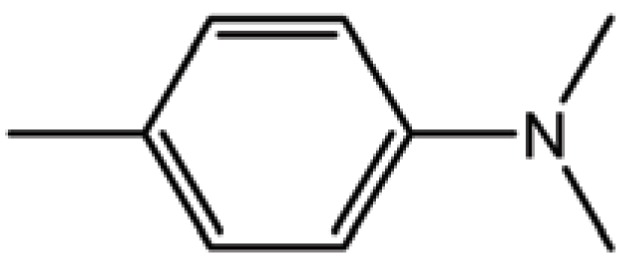	Br	9
**7**	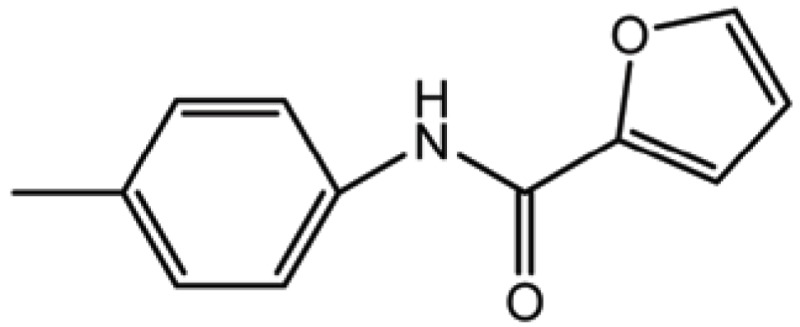	Br	0.62
**8**	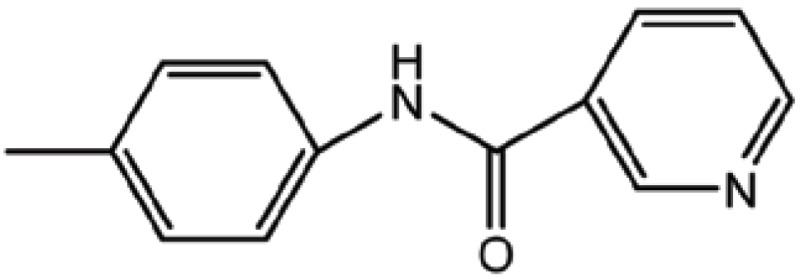	Br	0.82
**9**	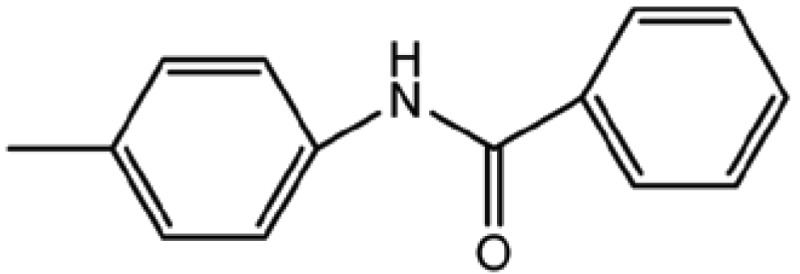	Br	0.6
**10**	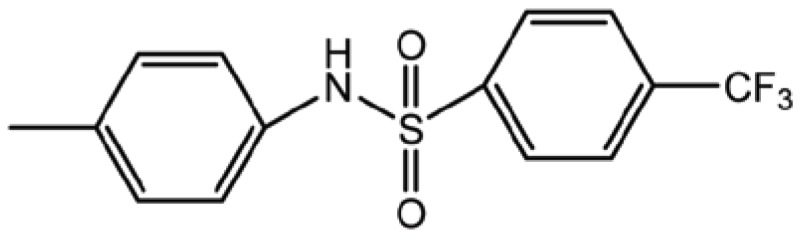	Br	0.2
**11**	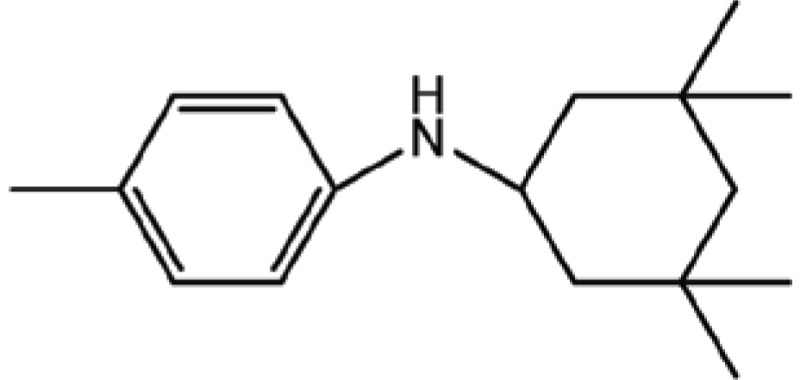	Br	0.25

**Table 2:** Structure and biological activity of compounds 12-16, 28-32 
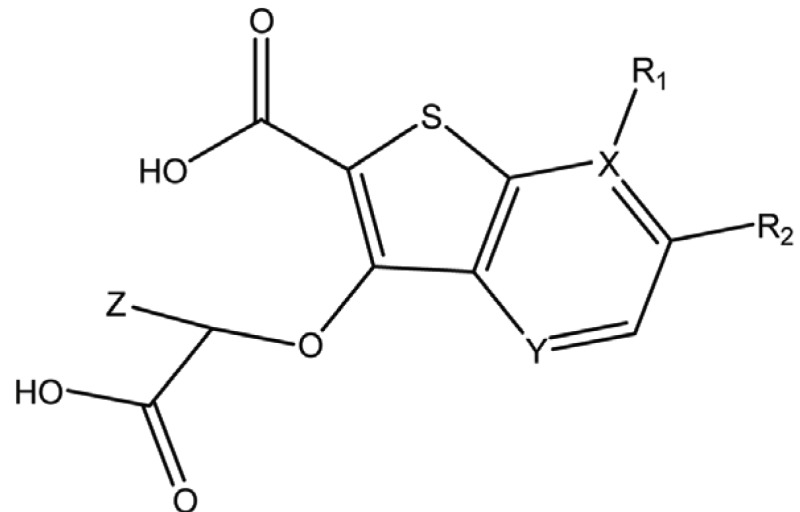

Compd. No.	X	Y	Z	R_1_	R_2_	Ki (μM)

**12**	-	N	H	H	H	77
**13**	-	-	H	H	Cl	61
**14**	-	-	F	H	Cl	52
**15**	-	-	H	H	Br	42
**16**	-	-	H	CH_3_	H	37
**28**	N	-	H	H	H	230
**29**	-	-	H	H	H	160
**30**	-	-	H	H	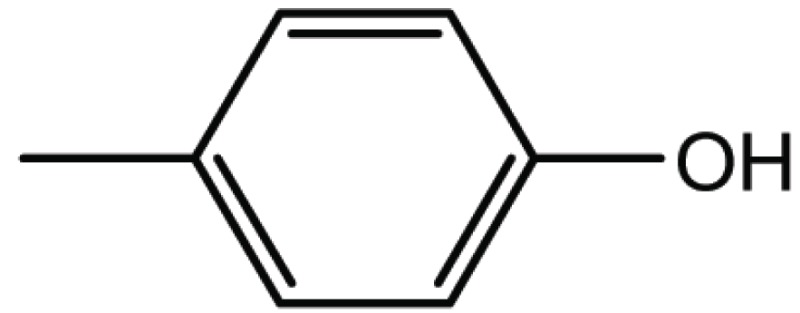	26
**31**	-	-	H	H	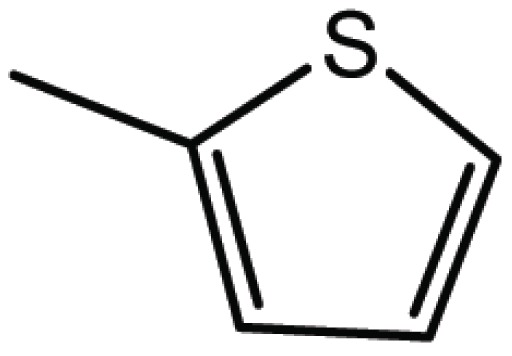	30
**32**	-	-	H	Cl	H	119

**Table 3:** Structure and biological activity of compounds 18-26, 33 
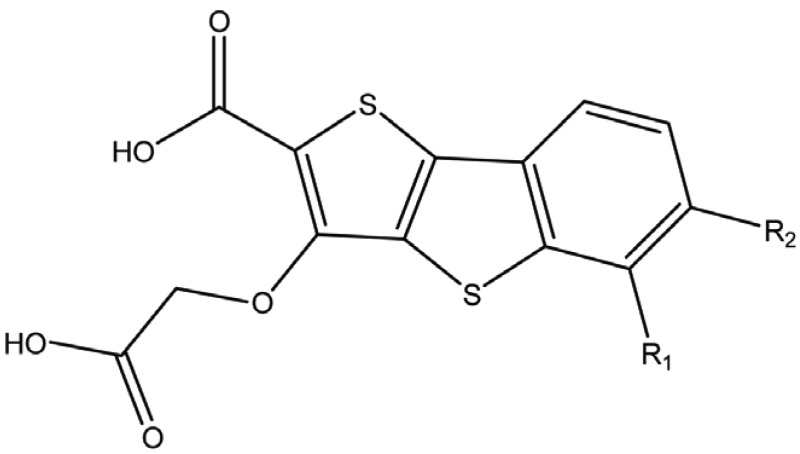

Compd. No.	R_1_	R_2_	Ki (μM)

**18**	H	H	9.2
**19**	Cl	H	10
**20**	H	Cl	3.5
**21**	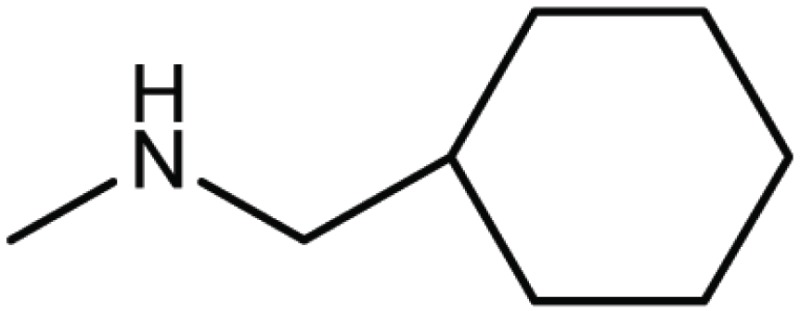	H	0.92
**22**	H	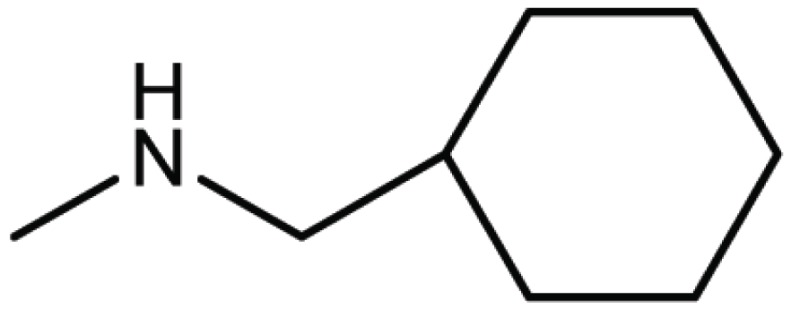	0.68
**23**	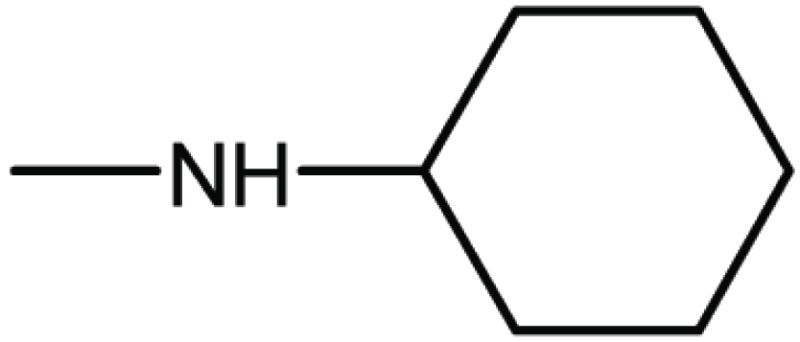	H	1.7
**24**	H	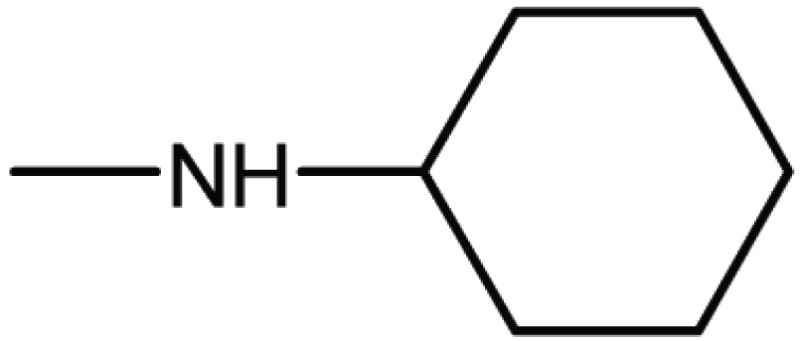	0.74
**25**	H	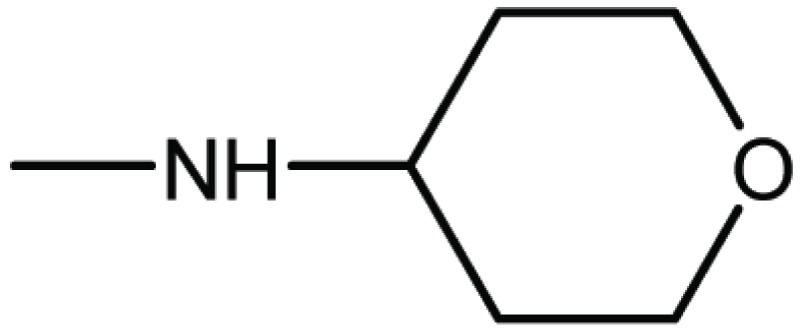	2.4
**26**	H	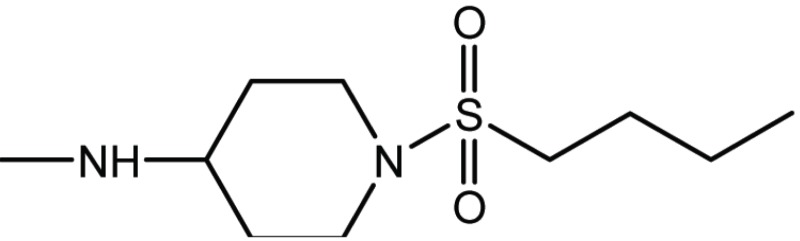	0.37
**33**	H	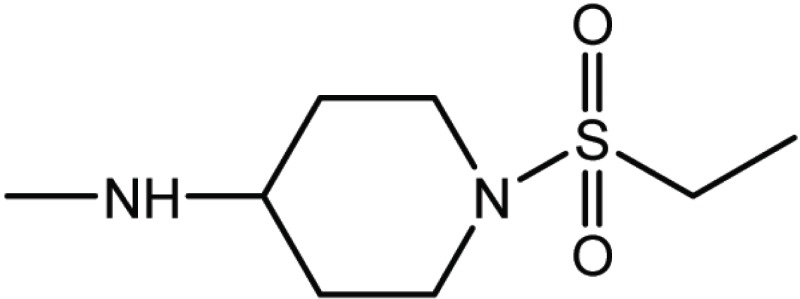	1.6

**Table 4:** Structure and biological activity of compounds 17, 27

Compd. No.	Compound	Ki (μM)

**17**	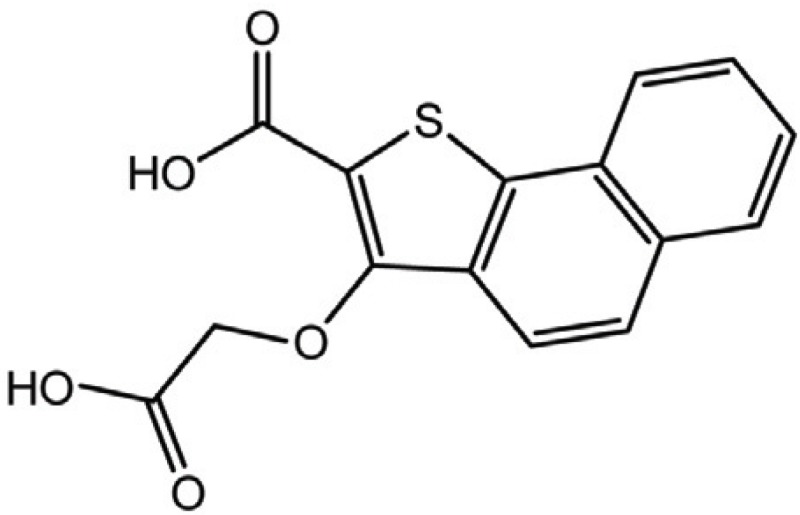	11
**27**	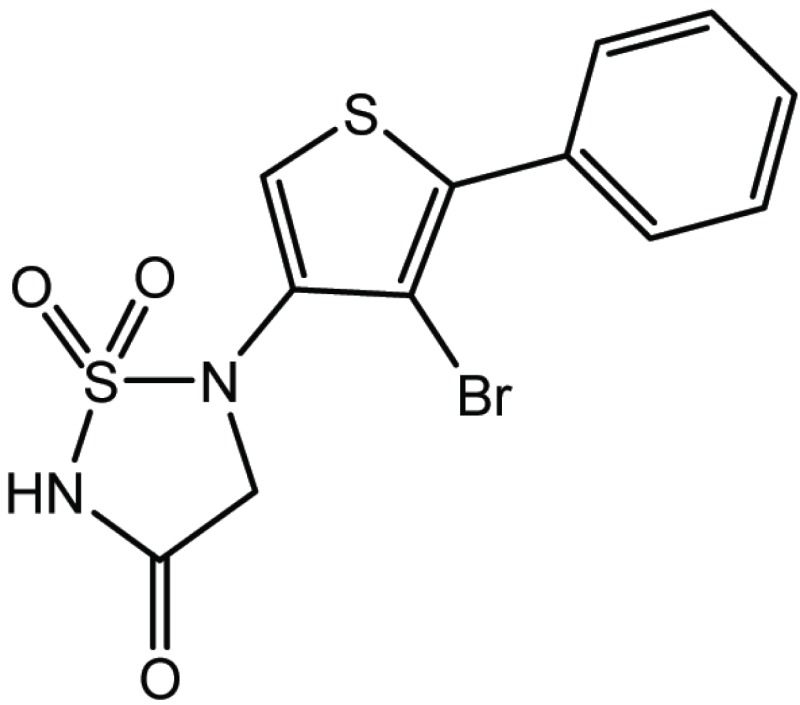	18

The Ki values employed in this work (varying from 0.2 to 160 μM), measured under the same experimental conditions, are acceptably distributed across the range of values. Thus, the data set is appropriate for the purpose of QSAR model development. The Ki values were transformed to pKi (-logKi) before being used as dependent variables in the QSAR investigations.

### Descriptor calculation and selection

Dragon 5.4, ChemOffice and TSAR 3.3 softwares were used to generate the physicochemical descriptors for the QSAR studies. Descriptors were obtained for the whole molecules. This procedure afforded 65 descriptors, which were subjected to the following selection strategy.

Reduced descriptors were obtained by discarding highly inter-correlated (r>0.8) descriptors and selecting descriptors that appeared with higher frequency in previous models. After that, the descriptors with r>0.9 between the activity and the descriptor were introduced to build the QSAR model. Also, descriptors possessing constant values as well as those with poor correlation to biological activity (r^2^<0.10) or that are more than 0.99 correlated were discarded.

### QSAR model development

The TSAR software was employed to systematically search for models of up to five variables that gave rise to multiple linear regression (MLR) models. Statistical measures used in stepwise multiple regression analysis are: n-number of compounds in regression, r-coefficient of correlation, r^2^-squared correlation coefficient, s-standard error or estimation and F-test (Fischer’s value) for statistical significance. Values of all the descriptors used in the study are given in Table 5.

**Table 5:** Physicochemical descriptors for test set and training set compounds

Compd No.	Pki	MW	log P	MR	Pol	Chi 0	Chi V0	Chi 1	Chi V1	Chi 2	Chi V2	Chi 3	Chi V3	RTI	BTI	WTI	MV	P

**1**	4.74	359.98	2.25	59.07	23.71	11.59	10.83	6.93	5.77	6.62	5.44	1.44	1.19	6.93	2.43	368	158.9	471.8
**2**	5.48	407.24	4.27	92.96	37.48	17.27	13.83	11.47	7.77	10.68	6.1	1.84	0.89	11.47	1.48	1340	242.1	698.4
**3**	5.3	397.2	2.89	85.48	34.43	16.56	13.24	10.97	7.42	10.33	5.88	1.84	0.88	10.97	1.49	1183	224.9	652.4
**4**	5.52	387.2	3.15	83.04	33.06	16.28	12.74	10.43	6.92	9.49	5.4	1.71	0.82	10.43	1.81	1083	232	651.3
**5**	3.8	281.08	1.54	51.14	20.66	10.72	8.832	6.52	4.77	6.1	3.65	1.25	0.55	6.52	2.34	311	142.7	421.3
**6**	5.05	400.24	3.56	90.97	36.09	17.15	13.28	10.81	7.19	10.22	5.66	2.01	0.88	10.81	1.82	1234	246	96.6
**7**	6.21	466.26	2.69	101.6	41.07	20.26	15.6	13.36	8.58	12.34	6.52	2.12	0.92	13.36	1.39	2272	269.7	788.2
**8**	6.09	477.29	2.74	107.56	43.36	20.97	16.13	13.86	8.86	12.69	6.7	2.12	0.93	13.86	1.38	2526	280.1	828.4
**9**	6.22	476.3	4.08	109.08	44.12	20.97	16.19	13.86	8.92	12.69	6.74	2.12	0.93	13.86	1.38	2526	286.9	834.2
**10**	6.7	580.35	4.6	119.36	46.94	25.26	19.02	15.79	11.3	16.19	9.25	4.42	1.62	15.79	1.41	3963	330.6	936.6
**11**	6.6	510.44	5.92	124.34	49.95	22.97	17.28	14.37	9.46	15.1	7.66	4.33	1.38	14.37	1.39	2972	366.8	987
**12**	4.11	253.23	0.85	57.19	23.93	12.41	8.779	8.09	4.96	7.49	3.79	1.32	0.51	8.09	1.86	504	153.9	468.7
**13**	4.21	286.69	2.32	64.28	26.63	13.28	10.03	8.49	5.61	8.12	4.44	1.61	0.67	8.49	1.87	592	172.6	510.5
**14**	4.28	304.68	3.12	64.04	26.68	14.15	10.41	8.9	5.8	8.61	4.62	1.77	0.71	8.9	1.94	673	178.4	517.4
**15**	4.38	331.14	2.59	67.37	27.74	13.28	10.83	8.49	6.02	8.12	4.85	1.61	0.77	8.49	1.87	592	176.8	525.1
**16**	4.43	266.27	2.25	65.57	26.6	13.28	9.33	8.50	5.27	8.02	4.1	1.52	0.58	8.50	1.88	581	176.9	512.2
**17**	4.96	302.3	2.76	76.84	31.76	14.98	10.83	10.08	6.27	9.39	4.85	1.59	0.64	10.08	1.55	878	194.8	578.4
**18**	5.04	308.33	2.8	73.89	31.12	14.28	11.12	9.58	6.81	9.05	5.71	1.59	0.84	9.58	1.58	773	184.2	561.6
**19**	5	342.77	3.36	78.49	33.06	15.15	12.32	9.99	7.4	9.58	6.31	1.79	0.99	9.99	1.6	877	196.2	597.5
**20**	5.46	342.77	3.36	78.49	33.06	15.15	12.32	9.97	7.4	9.68	6.31	1.88	0.99	9.97	1.59	891	196.2	597.5
**21**	6.04	419.51	4.27	108.77	45.11	19.67	15.07	13.54	9	12.44	7.16	1.93	0.96	13.54	1.25	2109	286.2	833.1
**22**	6.17	419.51	4.27	108.77	45.11	19.67	15.07	13.52	9	12.53	7.16	2	0.96	13.52	1.22	2221	286.2	833.1
**23**	5.77	405.49	3.84	104.09	43.27	18.97	14.57	13.04	8.75	12.1	7.02	1.93	0.95	13.04	1.28	1843	266.2	787.9
**24**	6.13	405.49	3.84	104.09	43.27	18.97	14.57	13.02	8.75	12.18	7.02	2	0.95	13.02	1.26	1941	266.2	787.9
**25**	5.62	407.46	2.09	101.21	42.08	18.97	14.48	13.02	8.66	12.18	6.95	2	0.95	13.02	1.26	1941	257.2	772.5
**26**	6.43	526.09	2.54	131.48	52.73	24.46	18.63	16.19	11.7	15.49	9.63	3.19	1.54	16.19	1.19	3983	341.7	1027.8
**27**	4.74	373.25	2.3	81.45	32.11	14.33	12.7	9.49	8.12	9.30	6.7	2.09	1.12	9.49	1.49	781	214	601.6
**28**	3.64	253.23	1.17	58.11	23.93	12.41	8.78	8.09	4.96	7.49	3.77	1.32	0.5	8.09	1.86	504	153.9	468.7
**29**	3.8	252.24	1.77	59.67	24.69	12.41	8.83	8.09	5.02	7.49	3.85	1.32	0.52	8.09	1.86	504	160.6	474.6
**30**	4.59	344.34	3.05	87.08	35.18	17.27	12.24	11.45	6.97	10.8	5.30	1.94	0.7	11.45	1.46	1383	224.4	663
**31**	4.52	334.37	3.42	83.26	33.8	15.69	12.12	10.56	7.31	9.82	5.76	1.65	0.74	10.56	1.49	1061	215.4	631.1
**32**	3.92	286.69	2.32	64.28	26.63	13.28	10.03	8.5	5.61	8.01	4.44	1.52	0.67	8.5	1.88	581	172.6	510.5
**33**	5.8	498.59	1.64	122.29	49.06	23.04	17.63	15.2	11.23	14.74	9.38	3.19	1.54	15.19	1.21	3284	309.5	947.7

Compound 1-27, training set; Compound 28-33, test set; MW, molecular weight; MR, molar refractivity; Pol, polarizibility; Chi 0-3, connectivity index of order 0-3, Chi V0-3, valence connectivity index of order 0-3; RTI, randic topological index; BTI, balaban topological index; WTI, wiener topological index; MV, molar volume; P, parachor.

## RESULTS

**Figure 1:**
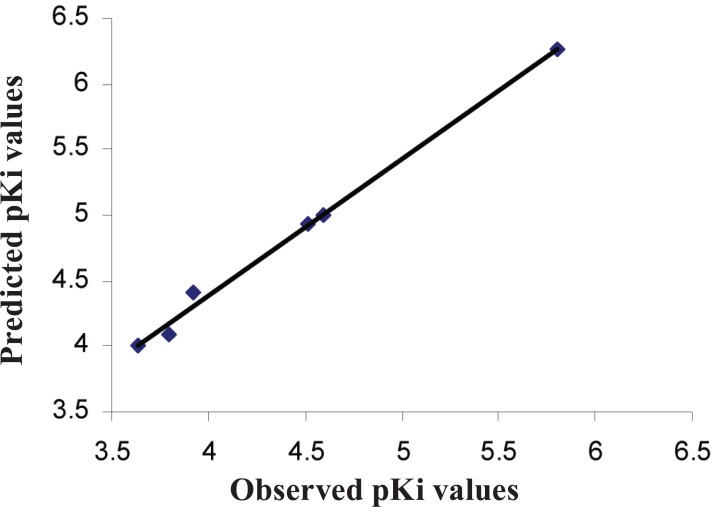
Plot of Observed Vs Predicted pKi values.

An important step in classical QSAR modeling is the selection of appropriate descriptors that are correlated to biological activity. Due to the large number of descriptors available, they were selected based on their biological activity and capability of producing MLR models with up to four descriptors with correlation (r^2^>0.8). This strategy had two goals: to build initial QSAR models that could shed light on structural features important for PTP1B binding, and to select a subset of the most correlated descriptors that could be further explored in QSAR model development. QSAR analysis from the 17 various descriptors generated many equations. Those which were statistically significant are shown in Table 6 along with their statistical parameters. The predictive power of the best QSAR model derived using the 27 training set molecules was assessed by predicting Ki values for 6 test set compounds (28-33), not used for QSAR model development. The external validation process can be considered the most reliable validation method, as cross-validation procedures may lead to very optimistic statistics (20). The results of the external validation are listed in Table 7, and the graphic results for the experimental versus predicted activities of both training set and test set are displayed in Figure 1.

**Table 6:** Selected QSAR equations along with their statistical parameters

Equation No.		Regression equation	Regression parameters			
			n	r^2^	S	F

1	pKi	= 0.065139(±0.058625)log P + 0.225557(± 0.106054)Pol - 0.07389(±0.040898)MR + 0.006774(±0.00605)MW - 0.04197(±0.249617)Chi V0 + 1.481678(±0.282038)	27	0.9416	0.2238	67.7094
2	pKi	= 0.097351(± 0.061619)log P + 0.115992(±0.078925)Pol - 0.04725(±0.035163)MR + 0.071835(± 0.112279)RTI + 0.203(± 0.086313)Chi V0 + 1.546012(± 0.284027)	27	0.9393	0.2282	64.9809
3	pKi	= 0.085488(± 0.06874)log P + 0.132932(± 0.095141)Pol - 0.04636(±0.055487)MR - 0.0004(± 0.00632)MV + 0.222009(± 0.083387)Chi V0 + 1.538643(± 0.292985)	27	0.9381	0.2304	63.6713
4	pKi	= 0.091019(± 0.057105)log P + 0.109206(± 0.075616)Pol - 0.03832(± 0.036033) MR + 0.000421(± 0.000405)P + 0.198072(± 0.082704)Chi V0 + 1.6109(±0.288627)	27	0.9411	0.2247	67.1453
5	pKi	= 0.067609(±0.055487)log P + 0.214947(±0.083334)Pol - 0.07168(±0.037871)MR + 0.005809(±0.001866)MW + 1.47848(±0.275111)	27	0.9415	0.2188	88.5405
6	pKi	=0.083143(±0.056701)log P + 0.136846(±0.070908)Pol - 0.04908(±0.034573) MR + 0.223251(±0.079215)Chi V0 + 1.534652(±0.27964)	27	0.9381	0.2251	83.3619
7	pKi	= 0.112048(±0.060279)log P + 0.089433(±0.022379)Pol + 0.004485(±0.001479) MW - 0.0069(±0.00429)MV + 1.653769(±0.234532)	27	0.9391	0.2232	84.8678
8	pKi	= 0.097624(±0.062941)log P + 0.032039(±0.033)Pol + 0.002562(±0.001305)MW + 0.096367(±0.111046)RTI + 1.819982(±0.216287)	27	0.9342	0.2320	78.1386
9	pKi	= 0.088246(±0.057114)log P + 0.047976(±0.013662)Pol + 0.002864(±0.001167) MW + 0.000584(±0.000386)P + 1.848973(±0.209555)	27	0.9384	0.2246	83.8003
10	pKi	= 0.109937(±0.061772)log P + 0.055949(±0.023558)Pol + 0.177963 (±0.064165)Chi V0 - 0.00447(±0.004002)MV + 1.675138(±0.241538)	27	0.9361	0.2288	80.5195
11	pKi	= 0.101603(±0.062654)log P + 0.01935(±0.033108)Pol + 0.121149(± 0.062267) Chi V0 + 0.084089(±0.113939)RTI + 1.787309(±0.224063)	27	0.9341	0.2323	77.9202
12	pKi	= 0.094707(±0.057169)log P + 0.031366(±0.019038)Pol + 0.131955(±0.054703) Chi V0 + 0.000544(±0.000389)P + 1.812581(±0.218223)	27	0.938	0.2254	83.154

**Table 7:** Prediction parameters of Equation 1

Compd. No.	Observed pKi	Predicted pKi	Residual

**28**	3.64	4.01	-0.37
**29**	3.8	4.09	-0.29
**30**	4.59	5	-0.41
**31**	4.52	4.93	-0.41
**32**	3.92	4.41	-0.49
**33**	5.8	6.26	-0.46

## DISCUSSION

Very low residuals in the test set model signify good predictability of the models. All other eleven models (equation 2-12) were found to possess good predictive power when subjected to external validation using test set compounds (data not shown). Good predictability as well as statistical significance (low s; high r^2^ and F values) turned out as peculiar features of the developed models satisfying primary requirements for a QSAR study.

Preliminary structure activity relationship studies revealed that monocyclic substituted thiophenes (compound 1-11) were more potent than fused thiophenes (compound 12-17). Also, an amide group or a secondary amino group increased activity significantly (as seen with compound 7-11, 21-26, 33) due to its involvement in H-bond interaction with Asp48 residue of the enzyme (19). Lipophilicity turned out to be an indispensable feature for showing PTP1B inhibition, which is consistent with the well-established interactions of ligand with hydrophobic Met258 side chain (17). In all the QSAR models developed, logP contributed significantly to the biological activity. The results also correlate with the fact that the most potent compounds (10 and 11) had the highest logP values (4.6 and 5.92, respectively). Additionally, the compound 5 with Ki value of 160 μM (pKi=3.8) had lowest value of logP, viz. 1.54. Thus, design of lipophilic compounds should be the strategy for future PTP1B inhibitors in this series. Various electronic parameters also contributed significantly to the biological activity. Polarizibility appeared to be affecting biological activity positively where as a negative effect of molar refractivity was observed. Effect of steric descriptors remained variable. Yet, valence connectivity indices, molecular weight, parachor and randic topological index were found very useful to generate statistically significant and predictive QSAR models.

## CONCLUSION

Twelve predictive QSAR equations were drawn with significant descriptors, the most important being lipophilicity, electronic and steric parameters, by multiple linear regression analysis. Excellent correlation with lipophilicity was observed, suggesting future design of additional lipophilic compounds. A little information was obtained about the effect of electronic descriptors on biological activity due to its variable effects in different models. As the models were predictive in nature, effect of electronic descriptors can be taken into account quite satisfactorily, when each model is taken individually. Models also gave an insight in terms of incorporation of group based on their electronic nature, i.e. electron release or withdrawal. Similarly, steric features were also well correlated with biological activity. Although it seemed difficult to judge its absolute effect, good predictive models were derived when steric features were taken along with different sets of lipophilic and electronic parameters.
